# Hospital Meetings

**Published:** 1905-04-15

**Authors:** 


### HOSPITAL MEETINGS.

### THE CITY ORTHOPÆDIC HOSPITAL.

Mr. W. G. Brighten presided at the annual general meeting of the City Orthopædic Hospital, Hatton Garden, on Wednesday, 5th inst. The report and statement of accounts, having been considered paragraph by paragraph, were adopted, various amendments being made to the former; the committee of hon. officers were re-elected, and the usual votes of thanks, including one of warm appreciation to Lord Mansfield (chairman), were passed.

Mr. J. Matthew Klenck said that, notwithstanding the anxiety and difficulty of the past year, the committee saw its way clear to extend its benefits to the crippled poor in the future as it had done in the past, and he was quite certain that the withdrawal of the grants of the Hospital Sunday Fund, on account of the refusal of the committee to amalgamate with the National and Royal Orthopædic Hospitals, had been indirectly of benefit, since it had placed the hospital more prominently before the public than ever before, the outside help having been such that, in his opinion, it was a matter of astonishment that the small amount contributed by that Fund for many years should be denied during the year under review. Having spoken with a degree of hope as to the grant not being withheld during the coming year, Mr. Klenck said the committee regretted that, although prior to the establishment of the Fund a considerable sum accrued to the hospital as a result of special sermons and collections, the hospital was debarred from participating in the proceeds now that they were given, in response to a general appeal, to a general fund. No satisfactory reason had ever been adduced for the withdrawal of the grant last year. He anticipated better results from the correspondence that had taken place and the publication of the pamphlet setting forth the position of the hospital, than the closing of the hospital doors. The hospital would not be closed, the committee would continue its good work, and he only hoped that funds would be given to the hospital to enable the beds at present empty to be filled. By treating the cripples in their childhood they were preventing them from becoming chargeable to their families and to the ratepayers in the future; and thanks to this and other orthopædic hospitals, the little cripple in the chimney-corner was now a thing of the past.

The Chairman said that while the refusal to amalgamate was, if any reason at all for withholding the grant, an improper one, he thought no good would be done by attempting to force the hands of the Sunday Fund.

A vote of thanks to the chairman concluded the proceedings.

### WEST LONDON HOSPITAL.

The annual meeting was held at the West London Hospital on Thursday, April 6th, the Duke of Abercorn being in the chair.

The report for 1904, with statement of accounts, was read and passed. The chairman entered at some length into the comparative expenditure of the West London Hospital and other similar institutions, and drew the attention of his audience to the gratifying fact that he believed the cost per bed at the West London Hospital was considerably lower than at any other hospital in the metropolis, being only £57 14s. 6d. The total cost of in-patients in 1904 was £9,410 5s. 11d., and that of the out-patients £2,014 9s. 9d.

His Grace further stated that, since the opening of the meeting, Mr. H. W. Casper had come forward and promised to endow a cot in perpetuity.

Mr. Lewis, the retiring chairman, made a short speech, and also touched on the financial state of the hospital, saying that in his long connection with the charity he had always seen that economy combined with efficiency and comfort had been the watchword of the hospital. Expenses were naturally very heavy and he hoped that subscriptions would come in from those at a distance as well as those in the neighbourhood. It was a poor district and unless help came from richer localities they would be in a bad way. However, there was nothing to complain of, subscribers and donors had been generous. King Edward's Fund had given them an annual subscription of £1,500 and a donation of £500. The Hospital Saturday Fund contributed £225 12s., and the Hospital Sunday Fund £1,015 16s. 8d.

Several votes of thanks were passed, including one to the retiring chairman, Mr. H. Lewis, one to the Ladies' Association, and one to his Grace the Duke of Abercorn for accepting the position of chairman.

### CENTRAL LONDON THROAT, NOSE AND EAR HOSPITAL.

The annual meeting was held at the above hospital on April 5, Captain Alfred Hutton being in the chair. The report for the year was adopted, one or two new rules being under discussion, such as that by which subscribers' letters are coming into force with regard to the admission of patients. It has been found absolutely necessary to enlarge the hospital, and the Council of King Edward's Fund have spoken very strongly on the subject, and have supported their opinion with a generous grant of £800 towards the building fund, in addition to the £500 given to the maintenance of the hospital. Mrs. Patrick Fraser has given £1,500, and the Trustees of Smith's Charity £500 for the same purpose. The necessary ground adjoining the hospital has been bought, and the work is already begun, but funds are urgently needed. The cost of the undertaking is £15,000, of which about £4,526 have been subscribed.

A vote of thanks to the treasurer, Captain Hutton, was proposed, seconded, and passed; he had tendered his resignation, but at the earnest wish of his colleagues on the committee he has consented still to act as chairman. Thanks were also tendered to the medical staff and to Mr. Calkin Lewis, to whose energy it is due in a great measure that the hospital has become incorporated.

